# Understanding the Process of Envelope Glycoprotein Incorporation into Virions in Simian and Feline Immunodeficiency Viruses

**DOI:** 10.3390/v6010264

**Published:** 2014-01-16

**Authors:** José L. Affranchino, Silvia A. González

**Affiliations:** Laboratorio de Virología, CONICET-Universidad de Belgrano (UB), Buenos Aires C1426BMJ, Argentina; E-Mail: jose.affranchino@comunidad.ub.edu.ar

**Keywords:** Simian immunodeficiency virus, feline immunodeficiency virus, Gag polyprotein, matrix protein, envelope glycoprotein, envelope incorporation

## Abstract

The lentiviral envelope glycoproteins (Env) mediate virus entry by interacting with specific receptors present at the cell surface, thereby determining viral tropism and pathogenesis. Therefore, Env incorporation into the virions formed by assembly of the viral Gag polyprotein at the plasma membrane of the infected cells is a key step in the replication cycle of lentiviruses. Besides being useful models of human immunodeficiency virus (HIV) infections in humans and valuable tools for developing AIDS therapies and vaccines, simian and feline immunodeficiency viruses (SIV and FIV, respectively) are relevant animal retroviruses; the study of which provides important information on how lentiviral replication strategies have evolved. In this review, we discuss the molecular mechanisms underlying the incorporation of the SIV and FIV Env glycoproteins into viral particles.

## 1. Introduction

Lentiviruses, members of the *Retroviridae* family, can be classified into two groups based on their cellular tropism and disease manifestations [1]. One group includes viruses that by infecting lymphocytes and cells of the monocyte/macrophage lineage cause an immunodeficiency syndrome. To this group belong the human immunodeficiency viruses types 1 and 2 (HIV-1 and HIV-2, respectively), simian immunodeficiency virus (SIV), feline immunodeficiency virus (FIV) and bovine immunodeficiency virus. The second group that corresponds to viruses that cause multiorgan disease and replicate predominantly in macrophages is represented by equine infectious anemia virus, maedi-visna and caprine arthritis-encephalitis virus. Despite their differences in cell tropism, lentiviruses share common features: long incubation periods before the onset of clinical symptoms, disease progression even in the presence of a strong host immune response and an almost invariably deadly outcome.

Lentiviruses, like all retroviruses, contain the *gag*, *pol* and *env* genes that encode the Gag polyprotein which is the major structural protein of the immature virion, the viral enzymes (protease, integrase and reverse transcriptase), and the envelope glycoprotein (Env), respectively [2]. Interestingly, the *pol* genes of nonprimate lentiviruses have evolved to contain an additional genetic element coding for dUTPase activity which prevents the incorporation of uracil into the reverse-transcribed viral DNA products [2]. In addition to these genes, lentiviral genomes exhibit a series of overlapping small open reading frames coding for regulatory proteins. In this regard, HIV and SIV are unique among lentiviruses because their genomes contain the largest number of auxiliary genes, namely *tat*, *rev*, *vif*, *nef*, *vpu*, *vpr* and *vpx*, the products of which are involved in the transcription of the viral genome, RNA processing, and in counteracting the antiviral activity of cell host restriction factors [3,4]. FIV lacks the *tat*, *nef*, *vpu*, *vpr*, or *vpx* regulatory genes of primate lentiviruses. Instead, FIV has a small open reading frame termed *orf-A* (or *orf-2*) encoding a 77-amino acid polypeptide which was initially reported to act as a transcriptional transactivator of the viral genome in a manner similar to HIV-1 and SIV Tat proteins [5]. However, later studies showed that Orf-A is a multifunctional protein influencing virus production, virus infectivity, and even cell cycle regulation [6,7]. Based on these findings, Orf-A appears to be similar to the primate lentiviral protein Vpr [7].

Lentiviruses assemble at the plasma membrane of the infected cells as a result of the multimerization of the Gag polyprotein into particles which then bud into the extracellular medium. The Gag polyprotein contains all the necessary information for the assembly and release of virions [8,9]. Indeed, the sole expression in cell cultures of the Gag precursor of HIV-1, SIV and FIV results in the formation of particles resembling immature virions [10,11,12]. Moreover, to facilitate the study of lentivirus assembly under controlled conditions, *in vitro* assembly systems have been developed using purified recombinant Gag proteins of HIV-1 [13,14], SIV [15] and FIV [16].

The generation of infectious lentiviral particles involves a series of steps: incorporation of the Env glycoprotein into virions, packaging of the viral RNA genome and processing of the Gag polyprotein by the viral protease into the matrix (MA), capsid (CA), and nucleocapsid (NC) proteins [8,9]. 

The incorporation of the Env glycoprotein into particles is a key step in the lentivirus replication cycle since this protein mediates virus entry into susceptible cells and therefore determines viral tropism and influences lentiviral pathogenesis. Here, we review our current understanding of Env packaging into lentiviral particles focusing on SIV and FIV, since this process has been extensively and comprehensively discussed for HIV-1 in recent articles [17,18,19].

## 2. Simian Immunodeficiency Viruses

SIV was first identified in captive macaques with immunodeficiency or lymphomas [20] and since then SIVs have been isolated from a large number of African nonhuman primate species without apparent disease [21]. SIV induces in macaques an immunodeficiency syndrome similar to human AIDS caused by HIV [22]. Moreover, SIV is morphologically, genetically and antigenically related to HIV. Because of these features, the SIV-macaque system is a useful model for the study of HIV infection and pathogenesis in humans [21].

SIVs are also highly relevant from an evolutionary perspective since HIV-2 originated from SIV of sooty mangabey monkeys (SIV_SMM_) [23,24] whereas the origin of HIV-1 has been traced to SIVcpz from the chimpanzee *Pan troglodytes* [25,26].

## 3. The SIV Env Glycoprotein

The SIV Env protein is synthesized in the rough endoplasmic reticulum as a single heavily glycosylated precursor (gp160) that is subsequently cleaved into the surface (SU, gp120) and transmembrane (TM, gp41) subunits by furin or furin-like proteases within the trans-Golgi network during its transport to the cell surface [27]. After gp160 processing, the gp120 and gp41 subunits remain associated by noncovalent bonds. Interestingly, the gp160 precursor oligomerizes into trimers within the endoplasmic reticulum and this trimeric structure is maintained in the mature gp120-gp41 complex that is present both at the plasma membrane of infected cells and on the virion surface [28]. 

### 3.1. SIV gp120

Incorporation of the gp120-gp41 complexes into virions is essential for SIV infectivity since gp120 sequentially binds to CD4 and a chemokine receptor present at the surface of the target cells, whereas the gp41 subunit induces the fusion of the viral and cellular membranes during virus entry. SIV, like HIV-1 and HIV-2, utilizes CD4 as primary receptor [29]. While the two biologically relevant coreceptors for HIV-1 are CCR5 and CXCR4, most SIV strains use CCR5 [30,31]. However, it has been reported that SIV is capable of using as coreceptors other molecules such as CCR3, CCR4, CCR8, CXCR6/STRL33/Bonzo, GPR1, GPR15/Bob and APJ [32]. The promiscuous use of coreceptors by SIV strains may be related to their ability to infect CD4-negative cells [33] and/or may be an evolutionary consequence of the broad variety of nonhuman primate species that are SIV natural hosts [21]. 

### 3.2. SIV gp41

**Figure 1:**
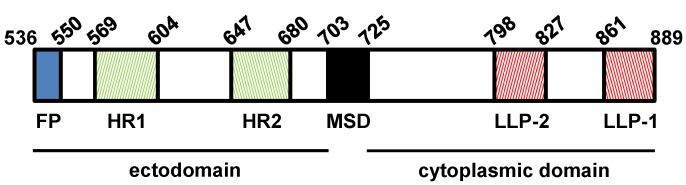
Domain organization of SIV_SMM-PBj_ gp41. The major gp41 structural regions are indicated: ectodomain, membrane-spanning domain (MSD) and cytoplasmic tail. The functional motifs within the ectodomain are depicted: the fusion peptide (FP) and the two heptad-repeats (HR1 and HR2). In the cytoplasmic domain, the two potential amphipathic α-helices are shown: lentiviral lytic peptides 2 and 1 (LLP-2 and LLP-1, respectively). Numbers correspond to the amino acid positions in the Env protein.

The gp41 subunit of SIV Env shares with the rest of the lentiviral TM glycoproteins a conserved structural organization: an ectodomain, a single membrane anchor region and a *carboxy*-terminal cytoplasmic domain (CD). The extracellular domain exhibits the *N*-terminal hydrophobic fusion peptide, two heptad-repeat regions (HR1 and HR2) that fold into α-helical coiled-coil structures and a membrane-proximal tryptophan-rich sequence [34,35] (Figure 1).

Binding of gp120 to CD4 and CCR5 triggers the exposure of the gp41 fusion peptide which inserts into the plasma membrane of the target cell [34]. This process is accompanied by the formation of a six-helix bundle that promotes the juxtaposition of the viral and cellular membranes thus allowing fusion to occur [35]. The six-helix bundle consists of three central HR1 regions wrapped in an outer layer of three antiparallel HR2 motifs [35].

All lentiviral glycoproteins, with the exception of that of FIV, contain unusually long CDs of 100–200 amino acids whose length contrasts with that of other retroviral Env proteins (less than 50 residues) [17,19]. The Env CD of the SIV_SMM-PBj_ strain [36] is 164-amino acid-long and exhibits a central and a *C*-terminal region predicted to fold into amphipathic α-helices designated as lentivirus lytic peptides 2 and 1 (LLP-2 and LLP-1, respectively) [37] (Figure 1). LLP-2 maps to Env residues 798–827 whereas LLP-1 is predicted to extend from amino acid 861 to residue 889 [38]. Synthetic peptides derived from these domains of HIV-1 gp41 have been shown to bind and perturb membranes [39,40] which led to the proposal that the LLP regions may associate with the inner leaflet of the lipid bilayer of both the plasma membrane and viral envelope. Recent biophysical experiments performed in HIV-1 have provided support to this hypothesis [41]. Moreover, it has been reported that the gp41 CDs of both HIV-1 and SIV bind to calmodulin which is a regulator of numerous cellular processes [42,43]. The association of the HIV-1 gp41 CD with calmodulin promotes Fas-mediated apoptosis and T-cell anergy which may be related to viral pathogenesis [44,45,46].

The SIV Env CD plays a critical role in modulating the levels of the gp120-gp41 complexes at the plasma membrane; Env is internalized by clathrin-mediated endocytosis through the interaction of CD motifs with the cellular adaptor protein complexes AP-1 and AP-2 [47,48]. One of the SIV CD signals involved in this process is the well-characterized membrane-proximal tyrosine-dependent motif GYRPV (residues 730–734 in SIV_SMM-PBj_ Env) [47,48] which is also present in HIV-1 Env [49]. Moreover, it has been shown that the SIV Env CD contains additional endocytosis and/or trafficking signals that regulate its surface expression on infected cells [48]. Although experiments performed in SIV_mac239_ Env indicated that the *C*-terminal dileucine motif does not participate in Env endocytosis [48], a later work demonstrated that the *C*-terminal dileucine in HIV-1_HXB2_ Env acts as an endocytosis signal [50]. These results suggest that SIV and HIV-1 Env proteins may exhibit certain differential trafficking signals [50].

It has been reported that passage of SIV_mac_ in human T-cell lines selects for variants with truncated Env CDs bearing only 18 amino acids [51,52] which is accompanied by an increased ability of these Env proteins to mediate membrane fusion and virus entry [53,54]. Interestingly, when these SIV mutants are inoculated in rhesus macaques, a rapid reversion to wild-type viruses encoding full-length Env proteins is observed [51], which highlights the requirement for an intact Env CD for SIV replication *in vivo*. In this regard, mutations within the immediate *C*-terminal region of the SIV_SMM-PBj_ CD drastically reduce the stability of the gp120-gp41 complex on the virion surface thereby severely impairing virus infectivity [55]. These results indicate that mutations at the extreme carboxyl end of SIV gp41 have long-range effects on its ectodomain which weakens the strength of the gp120-gp41 association [55].

## 4. The Matrix Domain of SIV Gag

**Figure 2:**
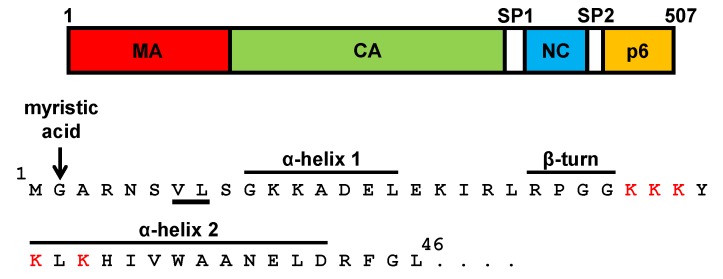
The amino terminal region of the matrix (MA) domain of SIV_SMM-PBj_ Gag. (Top) The domains of the SIV Gag polyprotein are depicted: matrix (MA, 135 amino acids), capsid (CA, 230 amino acids), nucleocapsid (NC, 52 amino acids), p6 (59 amino acids) and the spacer peptides SP1 (17 amino acids) and SP2 (14 amino acids). (Bottom) The amino acid sequence of the first 46 residues of the SIV MA is shown, indicating the α-helices 1 and 2, the β-turn, and the membrane-binding competent motifs: the myristic moiety that modifies the glycine at position 2 (arrow), the valine 7 and leucine 8 (underlined), and the polybasic region (amino acids 26–32, highlighted in red).

Processing of the SIV Gag polyprotein by the virus-encoded protease generates the mature proteins: MA, which forms the outer shell that is directly associated with the lipid viral envelope; CA, which forms the characteristic cone-shaped shell of the viral core; NC, which is associated with the genomic RNA within the viral core; and p6, which mediates virion budding [56]. In addition, two spacer peptides are generated by proteolytic cleavage of SIV Gag: SP1 and SP2, which separate the CA and NC, and the NC and p6 domains, respectively [56] (Figure 2).

During SIV assembly, the MA domain provides the primary determinants for the membrane targeting and association of the Gag precursor with the plasma membrane: the myristic acid which is cotranslationally added to the *N*-terminal glycine residue of the MA, the hydrophobic residues valine 7 and leucine 8, and a highly basic sequence located between MA residues 26 and 32 [11,57,58,59] (Figure 2). For HIV-1, it has also been demonstrated that the MA *N*-terminal region is responsible for Gag transport to and association with the plasma membrane [60,61,62,63]. Moreover, HIV-1 Gag membrane binding is regulated by a myristoyl switch mechanism whereby Gag multimerization together with binding of MA to phosphatidylinositol-(4,5)-biphosphate trigger the exposure of the myristate group [64]. Furthermore, it has been proposed that RNA binding to the HIV-1 MA polybasic region prevents premature nonspecific binding of Gag to cellular membranes prior to its association with the plasma membrane [65,66].

**Figure 3:**
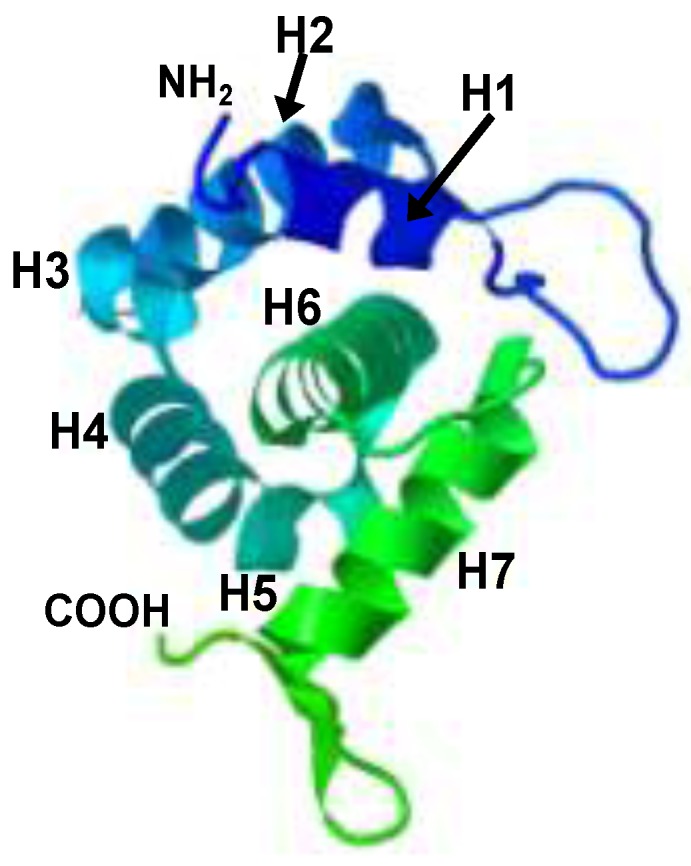
The crystal structure of the SIV MA (PDB 1ED1). A stereo view of the monomeric form of the protein (residues 6 to 119). The α-helices H1 to H7 are indicated as well as the amino and carboxyl termini of the molecule.

The crystal structure of the SIV MA shows that this molecule comprises five α-helices and two 3_10_ helices, and that the MA forms trimers which may represent a relevant intermediate during the process of SIV Gag assembly [67] (Figure 3). Comparison of the SIV MA crystal structure [67] with that of its HIV-1 counterpart [68] reveals that the latter also assembles into trimers and that both proteins exhibit a high degree of structure similarity.

Besides its role in Gag membrane association, the SIV MA participates in particle assembly as supported by the results from site-directed mutagenesis studies [11,69,70]. Indeed, the single amino acid substitution of alanine for MA cysteines 57 or 83 is sufficient to reduce particle production by 70% with respect to the assembly ability of wild-type SIV Gag [69]. Moreover, two domains in the SIV MA α-helix 6 were found to be essential for particle formation [70]. Helix 6 provides a hydrophobic core around which all the other helices are packed (Figure 3). Based on this, mutations in this region most likely alter the SIV MA structure thereby affecting the exposure of the interacting domains that participate in Gag multimerization [70].

## 5. Env Incorporation into SIV Virions

### The Interplay between the MA Domain of Gag and the Env CD

The first evidence that the MA domain of SIV Gag is involved in the packaging of the Env glycoprotein into virions was provided by the demonstration that the SIV MA is capable of self-assembling into lentivirus-like particles and that when the MA is coexpressed with the SIV Env protein, the MA-made particles incorporate the viral glycoprotein [11]. In addition, a mutation affecting residues located at the end of SIV MA helix 3 and the beginning of helix 4 was shown to abolish the association of Env with particles [70]. 

A detailed site-directed mutagenesis analysis of the SIV MA region spanning the two *N*-terminal α-helices was then performed so as to examine its role in Env incorporation and virus infectivity [71]. The domain targeted for mutagenesis is at an exposed side of the MA trimers which has been proposed to face the lipid bilayer and may therefore be in close proximity to the Env CD within the virion [67]. The results of this study indicated that amino acid substitutions at helix 1 or helix 2 abrogate Env incorporation into virions and virus infectivity [71] (Figure 2 and Figure 3). However, replacement of SIV MA arginine 22 and glycine 24 (Figure 2), alone or in combination, by hydrophobic amino acids increases the levels of virion-associated Env and enhances virus infectivity with respect to the wild-type virus [71]. Remarkably, the double amino acid substitution R22L/G24L augments virus infectivity eightfold when compared to wild-type SIV, an effect that correlates with a significant increase in Env incorporation into virions [71]. Interestingly, the R22L/G24L mutant virus replicates with faster kinetics and attains higher titers in CEMx174 cells than those of wild-type SIV [71]. Moreover, mutation R22L/G24L can even reverse the Env incorporation and virus infectivity defects imposed by mutations in helices 1 and 2 [71]. 

So how can these results be interpreted in the light of the available structural data? Based on the crystal structure of the SIV MA protein, it has been proposed that the trimers formed by this protein arrange into a lattice-like structure [67]. According to this model, helix 1 and the beginning of helix 2 lie at the perimeter of the holes formed by the SIV MA network with the β-turn structure comprising residues 22 to 25 projecting into these holes [67]. Thus, the location in the MA lattice of the region encompassing α-helix 1, the β-turn, and the beginning of α-helix 2 makes this MA domain a good candidate to interact with the Env glycoprotein CD [71]. Thus, the oligomerization of the SIV MA into trimers is a useful model to interpret the process of Env incorporation into virions. In this regard, work from independent groups suggests that MA trimerization is relevant for Gag multimerization and assembly. Indeed, it has been shown that: (i) the structure of the MA domain in a recombinant polypeptide corresponding to the first 283 *N*-terminal amino acids of HIV-1 Gag is essentially identical to that of the mature MA protein [72]; (ii) analysis of the multimeric state of HIV-1 Gag expressed in insect cells revealed that the MA domain forms trimers which contribute to a similar level of Gag oligomerization and that Gag trimerization driven by the MA domain is an intermediate stage in normal virion assembly [73]; (iii) the HIV-1 MA is trimeric in solution and mutations that affect trimerization also abrogate Gag assembly [74]; (iv) the myristoylated MA and myristoylated MA-CA proteins organize as hexamers of trimers upon interaction with lipid membranes [75] which suggests that, given that immature HIV-1 virions are formed by a lattice of Gag hexamers [76], the MA domain contributes to the high order of Gag organization during particle assembly [75]. In addition, it has recently been suggested that the trimeric arrangement of the HIV-1 MA may be a critical factor in allowing the incorporation of Env into the Gag lattice [77].

As mentioned above, most lentiviruses contain Env glycoproteins with exceptionally long CDs. In this regard, a key aspect of the lentiviral life cycle that remained to be elucidated was how the long Env cytoplasmic tail is accommodated into the assembling particles. This issue was addressed for SIV by two studies that investigated whether the gp41 CD plays any role in Env incorporation [38,78]. One of the studies showed that among a panel of short in-frame deletions introduced into the SIV Env CD, those targeting its *C*-terminal third portion (Env residues 832–889) block Env packaging into particles without affecting Env synthesis, processing, or transport to the cell surface [38]. Moreover, those mutations that impair Env incorporation also cause severe defects in virus infectivity [38]. The other report examined the effect of progressive truncation of the SIV gp41 CD on the biological properties of Env [78]. Removal of the first *C*-terminal 20 amino acids is sufficient to abrogate Env incorporation into particles and Env-mediated virus entry [78]. Further truncation of the SIV gp41 CD by 40 up to 80 residues also results in Env proteins that are incapable of associating with particles and mediating virus infectivity despite being efficiently expressed on the cell surface [78]. Interestingly, a mutant Env glycoprotein bearing a 64-amino acid-long CD is efficiently incorporated into particles but is unable to mediate virus entry into susceptible cells [78]. Remarkably, truncation of the SIV gp41 CD to 44 or 24 residues causes a drastic increase in both Env packaging into virions and virus infectivity with respect to wild-type SIV Env [78]. Overall, the data presented in these reports indicate that the *C-*terminal region of the SIV gp41 CD is essential for the incorporation of the full-length Env glycoprotein into virions [38,78]. 

It can be speculated that the mutant SIV Env glycoproteins with short CDs of 44 or 24 amino acids are incorporated into virions more efficiently than wild-type Env because they bypass the Gag-Env interactions that mediate the incorporation of Env glycoproteins with long cytoplasmic tails [78]. This notion is supported by three sets of evidence. First, the Env incorporation-defective phenotype caused by a mutation in the SIV MA domain of Gag is reversed by expression of an SIV Env glycoprotein with a short CD of 18 amino acids [70], an observation that has also been reported for HIV-1 [79,80]. Second, truncation of the Env CD to 44 amino acids restores viral infectivity in SIV_SMM-PBj_ MA mutants that are defective in the incorporation of the full-length Env protein [71]. Finally, long-term culture in CEMx174 cells of an Env incorporation-defective SIV_SMM-PBj_ mutant lacking Env residues 832–837 led to the emergence of two independent populations of revertant viruses encoding truncated gp41 CDs of similar lengths (52 and 48 amino acids) [81].

Notably, several site-directed mutagenesis and biochemical studies performed in HIV-1 also favor the concept that an association between the MA and the gp41 CD is necessary for Env incorporation into virions [82,83,84,85,86,87].

## 6. Physical Interaction between the SIV MA and the gp41 CD

The genetic and biochemical evidences discussed in the previous sections pointed to the notion that the interaction between the SIV gp41 cytoplasmic tail and the MA domain of Gag mediates the packaging of Env into virions. However, the physical interaction between these viral protein domains remained to be determined. In order to provide this functional proof, an *in vitro* association assay was developed to establish whether the SIV gp41 CD and MA protein are capable of a physical association [88]. The SIV gp41 CD was expressed in *Escherichia coli* as a fusion protein with *Schistosoma japonicum* glutathione S-transferase (GST), immobilized onto a glutathione-coupled Sepharose resin, and used as bait in GST pull-down assays [88]. To produce the prey protein, the SIV MA coding region was expressed in *E. coli* as fusion with intein, the protein splicing element of *Saccharomyces cerevisiae*, and the chitin-binding domain, and then purified by affinity chromatography. The intact SIV MA protein was recovered by treating the intein/chitin-binding domain-tagged MA bound to the resin with dithiothreitol, which induces the self-cleavage reaction at the *N*-terminus of the intein domain [88]. By using this pull-down assay strategy, the SIV MA was found to bind in a specific manner to the GST-CD_SIV_ fusion protein but not to beads coated with GST alone, demonstrating that the SIV MA and the gp41 CD are capable of establishing a direct physical interaction [88]. Binding of the SIV MA to the gp41 CD proved to be saturable with a dissociation constant of 7 × 10^−7^ M [88]. Furthermore, this association was blocked *in vitro* by mutations in either the MA or gp41 CD [88] that had previously been shown to interfere *in vivo* with Env incorporation into virions [71,78].

Interestingly, the SIV MA was capable of associating with the HIV-1 gp41 CD but not with that of the distantly related lentivirus FIV [88]. In addition, it was shown that the HIV-1 MA binds to the SIV gp41 CD with an efficiency similar to that observed for the HIV-1 MA/HIV-1 gp41 CD association [88]. These findings are in keeping with: (i) the possibility of generating infectious replication-competent SIV-HIV chimeric viruses (SHIVs) expressing SIV Gag and HIV-1 Env [89]; and (ii) the fact that Env-defective HIV-1 virions can be efficiently pseudotyped with SIV Env [38,53]. 

It has been proposed that the cellular protein TIP47 (Tail-interacting protein of 47 kDa) acts as a connector between the HIV-1 Gag and Env proteins during virion assembly [90]. However, in the GST pull-down assays described above, the addition of recombinant TIP47 resulted in its association with HIV-1 gp41 CD but did not enhance the *in vitro* binding of the HIV-1 MA with the gp41 CD [88]. Likewise, the presence of TIP47 had no effect on the *in vitro* interaction between the SIV MA and the gp41 CD proteins [88]. In support of these data, it has been recently reported for HIV-1 that TIP47 is not involved in the process of Env incorporation into virions [91]. In summary, the pull-down assays using recombinant SIV MA and Env CD proteins strongly support the notion that the interaction between these viral protein domains is an essential step in the process of SIV Env incorporation into virions.

## 7. Current Knowledge on the Process of FIV Env Incorporation into Virions

FIV is a lentivirus that induces in domestic cats an AIDS-like disease similar to that caused by HIV-1 in humans [92]. FIV is intensely studied since it represents an important cat pathogen and is a useful model for HIV-1 infections in humans. FIV infects a broad range of cell types such as CD4^+^ and CD8^+^ lymphocytes, B lymphocytes and macrophages [93,94,95]. In contrast to HIV-1 and SIV, FIV utilizes CD134 as primary receptor instead of CD4 [96,97]. However, the FIV Env glycoprotein binds to the chemokine receptor CXCR4 which is also used as coreceptor by T-tropic HIV-1 strains [98]. 

**Figure 4:**
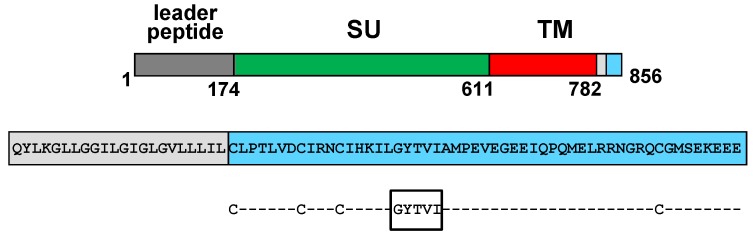
Schematic diagram of the feline immunodeficiency virus (FIV) Env glycoprotein. (**Top**) The leader peptide and the surface (SU) and transmembrance (TM) subunits are shown. The three major regions that constitute the TM are indicated: ectodomain (red box), membrane-spanning domain (grey box) and the cytoplasmic tail (light-blue box). Amino acid numbering corresponds to the Env protein of the Petaluma isolate. (**Bottom**) Amino acid sequences of the membrane anchor and the cytoplasmic domain of FIV Env. The four highly conserved cysteines as well as the tyrosine-based endocytosis motif within the cytoplasmic tail are highlighted below the sequence.

The FIV Env glycoprotein is initially synthesized as a precursor of 150 kDa which is processed into a 130 kDa species by removal of an unusually long leader peptide of 174 residues [99]. The 130 kDa Env precursor is further cleaved into the SU (gp95) and TM (gp38) subunits [99] (Figure 4) which perform similar functions to those of their HIV-1 and SIV counterparts. FIV SU mediates binding to the cell surface receptors [100], whereas the TM promotes fusion of the viral and cellular membranes during virus entry [101,102]. It has recently been demonstrated that the V3 domain of the FIV SU is essential for virion association with CXCR4 [103,104,105]. Of note, replacement of the V3 domain in the FIV Env glycoprotein with the V3 loop of a T cell-tropic HIV-1 results in a chimeric SU that not only binds efficiently to CXCR4 but is also capable of promoting CXCR4-dependent cell-to-cell fusion [105]. 

The FIV TM exhibits typical lentiviral organization: an ectodomain, a single membrane anchor, and a CD. However, the FIV Env is unique among lentiviral glycoproteins in that its CD bears only 53 amino acids [99] (Figure 4). The relevance of the relatively short TM cytoplasmic tail to FIV Env-mediated viral functions has been investigated by analyzing the biological properties of a series of FIV Env proteins progressively shortened from the *C*-terminus [106]. Deletion of 5 or 11 residues from the Env *C*-terminus does not affect Env surface expression, fusion activity, or Env incorporation into virions, whereas further truncation by 17, 23, or 29 amino acids impairs cell-to-cell fusion [106]. Interestingly, mutant FIV Env glycoproteins with CDs of only 18 or 12 residues are more fusogenic than wild-type Env and are incorporated into virions at levels higher than those of their wild-type counterpart [106]. The phenotype of these FIV Env mutants may be explained by the fact that they lack the tyrosine-based endocytosis motif GYTVI (CD residues 18 to 22; Figure 4) which may result in increased levels of biologically active SU at the cell surface due to a reduced rate of glycoprotein endocytosis [106]. Alternatively, shortening the FIV Env CD to 18 or 12 residues may cause structural changes in the TM ectodomain which may enhance fusion efficiency or kinetics [106]. In support of the concept that the TM CD modulates Env structure and function, it has previously been reported that deletions at the TM *C*-terminus affect the fusion kinetics of HIV-1 Env [107], the exposure of HIV-1 gp120 epitopes [108,109], and the stability of the gp120-gp41 complex on SIV virions [55].

Interestingly, an FIV Env exhibiting a short CD of only 6 amino acids is incorporation-competent but is incapable of mediating cell-to-cell fusion [106]. The phenotype of this FIV Env mutant is reminiscent of that of the SIV Env glycoprotein the CD of which was shortened to 64 residues. This SIV Env mutant is incorporated into virions albeit in a biologically inactive form as judged by its inability to mediate virus infectivity [78]. The nonfunctional nature of these mutant FIV and SIV Env proteins may be attributed to either their failure to trimerize or, alternatively, to their oligomerization into misfolded Env complexes. In support of this view, it has been shown that infectious HIV-1 virions exhibit on their surface a small number of inactive Env proteins [110] which have been characterized as gp120-gp41 monomers [111].

It has recently been demonstrated by means of Click chemistry assays that the four highly conserved cysteine residues within the FIV Env cytoplasmic tail (CD amino acids 1, 8, 12, and 45) are modified by palmitoylation [112] (Figure 4), a form of fatty acylation that plays a critical role in the regulation of protein function and cell signaling [113]. Of note, the double amino acid substitution C1S/C8S as well as the triple mutation C1S/C8S/C12S abrogate both FIV Env fusogenicity and Env incorporation into virions without affecting Env synthesis, processing or cell surface expression [112]. 

For HIV-1, it has been shown that a substantial fraction of the Gag polyprotein is associated with lipid rafts and that these membrane microdomains are involved in Gag assembly and release [114,115,116]. One distinctive property of lipid raft-associated proteins is their insolubility in Triton X-100 at 4 °C [117]. Interestingly, it was found that the virion incorporation-defective FIV Env protein C1S/C8S/C12S is as resistant to Triton X-100 extraction at 4 °C as the wild-type FIV Env, indicating that this mutation does not prevent Env localization in lipid rafts and also suggesting that this mutant glycoprotein colocalizes with FIV Gag [112]. 

In the case of HIV-1 and SIV, their gp41 CDs have also been found to be palmitoylated [118]. However, in contrast to the FIV TM CD which, as described above, is S-acylated at cysteines that are in close proximity to the membrane anchor [112], addition of palmitate to the HIV-1_HXB2_ Env protein takes place at cysteines 764 and 837 which are located at a distance of 59 and 132 amino acids, respectively, from the cytoplasmic side of the proposed membrane-spanning domain [118]. With respect to SIV, palmitoylation targets cysteine 787 in the SIV_mac239_ Env glycoprotein which lies at a distance of 71 residues from the *C*-terminus of the membrane anchor [118]. The role that palmitoylation of the SIV gp41 CD plays in the viral life cycle has not been clearly defined. For HIV-1, contradictory results have been reported on how removal of the gp41 palmitoylation sites affects Env functions. Indeed, Rousso and colleagues [119] observed that mutation of both Env cysteines 764 and 837 in HIV-1_HXB2_ decreases Env association with lipid rafts, Env incorporation into virions, and viral infectivity. Using HIV-1_NL4-3_, which only conserves cysteine 764, Bhattacharya *et al.* [120] showed that Env biological properties are only impaired when cysteine 764 is replaced by serine or alanine, whereas substitutions such as C764F or C764Y restore virus infectivity. In marked contrast, Chan *et al.* [121] reported that cysteine-to-serine mutations at HIV-1 Env residues 764 and 837 do not affect Env cell surface expression, Env incorporation into virions, or virus replication in CD4^+^ T cells.

The role in virus morphogenesis of the MA domain of FIV Gag has been addressed in a study characterizing the biosynthesis, transport to the cell surface, and particle assembly phenotype of a series of Gag polyproteins carrying MA mutations [12]. This report identified FIV MA domains involved in either Gag transport/association with the plasma membrane or particle formation [12].

The structural and functional relationships between the MA proteins of FIV and SIV were studied by characterizing the assembly and Env incorporation phenotypes of chimeric viruses in which the MA domain of one virus was partially or fully replaced by the equivalent region of the other virus [59]. A chimeric SIV provirus containing the central and carboxy-terminal regions of the FIV MA assembled into particles as efficiently as wild-type SIV [59]. However, the resulting virions were noninfectious since they were found to be incapable of recruiting the SIV Env glycoprotein [59]. Furthermore, when the entire MA domain of SIV Gag was replaced by that of FIV, the chimeric virus did not assemble into virions due to inefficient membrane binding of the chimeric Gag protein [59]. Interestingly, the assembly-defective phenotype of this chimeric virus could be reversed either by increasing the number of basic residues in the FIV-derived MA domain or by coexpression with wild-type SIV Gag [59]. Notably, a chimeric FIV provirus expressing the SIV MA not only assembled into virions as efficiently as wild-type FIV, but also replicated in feline T cells in a wild-type manner [59]. This study therefore demonstrates that the SIV MA can functionally replace the FIV MA, whereas the latter in the context of SIV Gag abrogates virion assembly. 

Despite the available information on the FIV MA functions, a key issue that remains to be elucidated is whether it participates in the incorporation of the Env glycoprotein into virions.

## 8. Conclusions

In addition to its role in membrane targeting and association of the Gag polyprotein with the plasma membrane, the SIV MA domain participates in the process of Env incorporation into virions. Indeed, mutations within the SIV MA region spanning the amino-terminal ɑ-helices H1 and H2 confer to Gag a differential ability to associate with the Env protein, thereby modulating either positively or negatively not only the levels at which Env is packaged into virions but virus infectivity as well [71]. Likewise, truncations or in-frame deletions affecting the carboxy-terminal third of the SIV gp41 CD abrogate Env incorporation into particles [38,78,81]. Of note, an *in vitro* binding assay using recombinant proteins expressed in bacteria led to the demonstration of a physical interaction between the SIV MA and the gp41 CD in the absence of other viral or cellular proteins [88]. Collectively, the results of these *in vivo* genetic and biochemical studies together with those of the *in vitro* binding assays strongly support the notion that the association between the SIV MA and the gp41 CD is a necessary step for the incorporation of the Env glycoprotein into virions.

In FIV, despite the relatively short length of the TM CD, certain amino acid substitutions within this domain have been shown to prevent Env association with viral particles without affecting Env trafficking or Env localization to lipid rafts, which indicates that the FIV TM CD is involved in Env incorporation [106,112]. Further studies will be necessary to establish whether the MA domain of the FIV Gag precursor plays any role in Env packaging into virions, which will certainly provide relevant information on how this essential process of the lentiviral life cycle has evolved.
